# The microRNA feedback regulation of p63 in cancer progression

**DOI:** 10.18632/oncotarget.3020

**Published:** 2015-01-23

**Authors:** Changwei Lin, Xiaorong Li, Yi Zhang, Yihang Guo, Jianyu Zhou, Kai Gao, Jing Dai, Gui Hu, Lv Lv, Juan Du, Yi Zhang

**Affiliations:** ^1^ Department of General Surgery, The Third XiangYa Hospital of Central South University, Changsha, Hunan 410013, P.R. China; ^2^ Department of General Surgery, The XiangYa Hospital of Central South University, Changsha, Hunan 410013, P.R. China

**Keywords:** p63, microRNA, cancer, feedback

## Abstract

The transcription factor p63 is a member of the p53 gene family that plays a complex role in cancer due to its involvement in epithelial differentiation, cell cycle arrest and apoptosis. MicroRNAs are a class of small, non-coding RNAs with an important regulatory role in various cellular processes, as well as in the development and progression of cancer. A number of microRNAs have been shown to function as transcriptional targets of p63. Conversely, microRNAs also can modulate the expression and activity of p63. However, the p63–microRNA regulatory circuit has not been addressed in depth so far. Here, computational genomic analysis was performed using miRtarBase, Targetscan, microRNA.ORG, DIANA-MICROT, RNA22-HSA and miRDB to analyze miRNA binding to the 3′UTR of p63. JASPAR (profile score threshold 80%) and TFSEARCH datasets were used to search transcriptional start sites for p53/p63 response elements. Remarkably, these data revealed 63 microRNAs that targeted p63. Furthermore, there were 39 microRNAs targeting p63 that were predicted to be regulated by p63. These analyses suggest a crosstalk between p63 and microRNAs. Here, we discuss the crosstalk between p63 and the microRNA network, and the role of their interactions in cancer.

## INTRODUCTION

The regulation of gene expression is implicated in multiple molecular mechanisms, which include transcriptional, translational, and post-translational regulatory mechanisms [1, 2]. In addition, the modulation of gene expression by transcription factors and microRNAs (miRNAs) is implicated in the control of gene expression [3–5].

**Figure 1:**
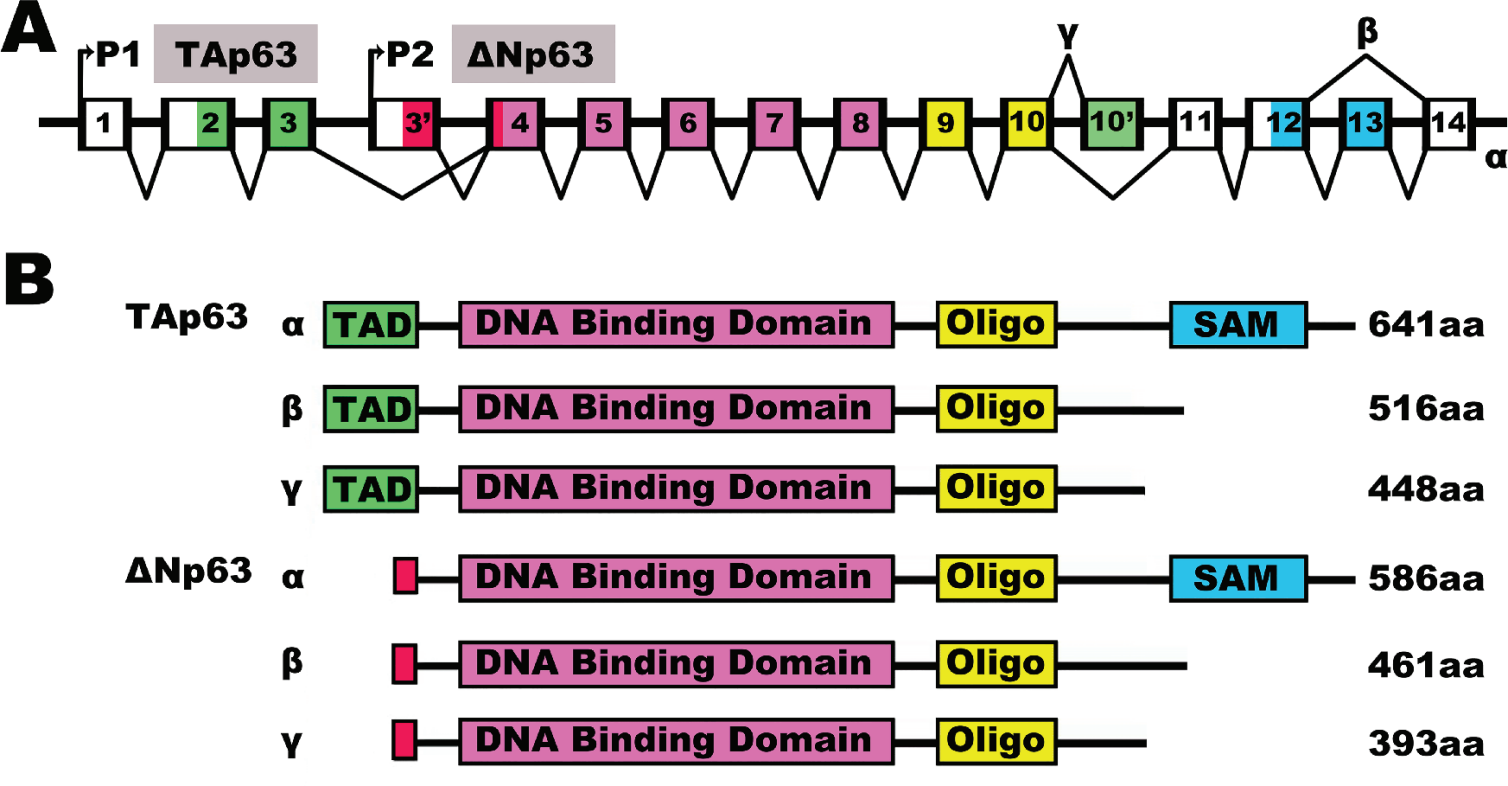
The human p63 splicing isoforms The human p63 splicing isoforms. **(A)** Schematic representation of intron/exon structure of the human p63 gene. The p63 gene has two promoters with the P1 promoter coding for TA (transactivation) isoforms and the P2 promoter coding for N-terminally truncated (ΔN) isoforms. Introns are depicted in white, while exons are colored according to the functional domains (see B). P63 genes express three splice variants and contain different internal promoters. **(B)** Schematic representation of the human p63 protein splicing variants. Various human p63 variants encoded by the p63 gene with indicated amino acid identity above the TA, DNA binding and oligomerization domain. TAD, Transactivation domain; ΔN, N-terminally truncated variants; Oligo, Oligomerization domain; SAM, Sterile alpha motif.

P63 is a member of the p53 gene family of transcription factors [6, 7]. Like other members of the p53 family [8], the p63 gene generates multiple protein isoforms with distinct properties from two distinct promoters [9]. The transcription of promoter 1, located upstream of exon 1, results in the expression of p63 transcripts containing the N-terminal transactivation domain (TA isoform). Alternatively, transcription from promoter 2, located in an intron, generates p63 transcripts with an N-terminal truncated isoform (ΔN isoform) (Figure 1A) [10]. Moreover, both TAp63 and ΔNp63 can be alternatively spliced at the C-terminal sequence to generate three isoforms named α, β and γ (Figure 1B) [11]. TAp63 is related to cell-cycle arrest and apoptosis and, similar to p53 [12], low levels of TAp63 are detected in various types of cancer tissues [13, 14]. Conversely, ΔNp63 has been implicated in cell proliferation and cell adhesion [15, 16], and this isoform is usually overexpressed in tumor tissues [17–19]. Thus, p63 exhibits isoform-specific expression and functions in human cancer [20, 21].

Although p63 has been shown to closely associate with cancer development, the underlying molecular mechanisms have not been fully elucidated. In fact, many studies indicate that transcriptional regulation of p63 is one of the most important mechanisms in various tumor processes by triggering the expression of oncogenes [22, 23]. TAp63 is a proven transcription factor that activates downstream targets with an N-terminal transactivation (TA) domain. ΔNp63 was originally believed to be a dominant negative transcriptional repressor because it lacks the N-terminal TA domain. However, it has been reported that ΔNp63 indeed has transcriptional activity due to the presence of alternative transactivation domains [24, 25]. Furthermore, an increasing number of studies have revealed that p63 activation is able to modulate the expression of miRNAs, which in turn can dampen the expression of hundreds of genes. Indeed, TAp63 may be able to inhibit metastasis by directly activating DICER and miR-130b [26], and ΔNp63 may enhance cell migration and metastasis by directly activating miR-205 [27].

miRNAs are 18- to 24-nucleotide non-coding RNA molecules [28]. It regulates gene expression at the post-transcriptional level by targeting to sites in the 3′ untranslated region (UTR) of messenger RNA (mRNA) and leading to mRNA degradation or inhibition of translation. It has been reported that miRNAs may regulate over 60% of mRNAs [29, 30]. miRNAs have been implicated in cellular differentiation, development, proliferation and apoptosis [31]. However, in cancer, imbalances in miRNA expression lead to the malfunction of these processes [32–34]. Thereby, miRNAs are likely to play their decisive role in tumor progression, diagnosis and prognosis [35–37].

miRNAs are canonically produced through a multistep process and transcription factors are recognized as key regulatory points for miRNA production [38]. Abnormal expression of transcription factors, such as p63, can promote miRNA imbalance and tumor progression [39, 40]. In fact, miRNAs can be transcriptionally or non-transcriptionally regulated by p63 [41]. Alternatively, the transcription factor activity of p63 has been shown to function downstream of miRNAs. miRNAs can regulate p63 by direct suppression of p63 expression or alternative indirect mechanisms [42]. These findings suggest that miRNAs are important components of the p63 network. Here, we provide an overview of the potential roles of the crosstalk between p63 and miRNAs in tumor suppression and cancer prevention.

### Alteration of p63 expression in human cancer

The human p63 gene belongs to the p53 family and plays a crucial role in maintaining genomic stability and suppressing tumor formation [43]. Although it has been suggested that p63 is involved in tumorigenesis, its role in either cancer initiation or progression is controversial [44]. Initially, several thorough analyses of p63 expression in cancer tissues demonstrated that p63 was often upregulated at the transcriptional level in squamous cell carcinoma [45, 46]. This strong evidence supported an oncogenic role for p63 in tumorigenesis despite its homology with p53, which is a classic tumor suppressor that originally suggested a tumor suppressive role for p63 [47]. These apparently contradictory results were surprising.

The p63 gene contains two promoters and produces two diametrically opposite groups of isoforms: those containing the transactivation (TA) domain (TAp63), and those lacking it (ΔNp63) [48]. ΔNp63 is transcribed from the second transcriptional start site within the p63 gene with three different C-termini termed α, β and γ, and it only contains a DNA binding domain and an oligomerization (oligo) domain. Because ΔNp63 isoforms lack the N-terminal TA domain, they were originally believed to be dominant negative transcriptional repressors. However, it has become clear that they also have transcriptional activity attributed to other transactivation domains [49]. ΔNp63 has also been implicated in cell proliferation and cell adhesion [50, 51]. Crook T et al. showed that ΔNp63 was overexpressed in 25 primary nasopharyngeal carcinomas [52]. Various studies have proven that ΔNp63 is an oncogene [53, 54]. Interestingly, Cui R's study suggested that both TAp63 and ΔNp63 isoforms are specifically upregulated at the transcriptional level in squamous cell carcinoma, and ΔNp63 was the predominant isoform expressed at the protein level [55]. These findings prompted us to investigate the roles of TAp63 in cancer.

TAp63 is transcribed from the first transcriptional start site within the p63 gene and contains the transactivation domain and three different C-termini termed α, β and γ [56]. The highlighted regions of TAp63 are the transactivation (TA) domain, DNA binding domain and oligomerization (oligo) domain [57]. TAp63α and ΔNp63α proteins contain an additional region referred to as the sterile alpha motif, or SAM domain, that is not found in p53 [58]. The SAM domain is a protein-protein interaction domain that was initially implicated in developmental processes and is involved in other processes such as apoptosis, transcription, and adhesion [59]. The most significant difference between TAp63 and ΔNp63 is in the transactivation (TA) domain [60]. Similar to p53, TAp63 expression can be induced in response to cellular stress. It is also part of the DNA damage response function, which induces cell-cycle arrest and apoptosis [61, 62]. Therefore, TAp63 is usually detected at low levels in cancer tissue [63, 64]. Accumulated studies suggested that TAp63 exerts different effects in carcinogenesis [65–67].

The role of p63 in tumorigenesis is very complex. Thus, more work is needed to explain how the differential expression of the p63 isoforms could influence this process. Considering the fact that all p63 isoforms contain a DNA binding domain and are able to bind to thousands of gene promoters, studying their interaction with regulatory genes may not only increase our understanding of the role of p63 in tumorigenesis, but could also open the door to the development of innovative diagnostic procedures and targeted therapies.

### miRNAs and their role in tumorigenesis

miRNAs are small, non coding RNAs with a length of 18 to 24 nucleotides [68]. They play an important role in all biological processes by post-transcriptionally regulating gene expression [69]. To date, approximately 1,881 precursors and 2,588 mature miRNAs have been identified in humans (Homo sapiens, miRbase release 21) [70, 71]. Computational and experimental approaches indicate that a single miRNA may repress more than one hundred mRNAs by annealing to the 3′UTR of gene transcripts [72]. Therefore, miRNAs are considered to regulate approximately 60% of human protein-coding genes [73], and constitute one of the most abundant classes of gene-regulatory molecules in animals by leading to mRNA degradation or the inhibition of translation [74]. It is noteworthy that recent evidence suggests that miRNAs exert a uniquely important role in cellular differentiation, development, proliferation and apoptosis [75, 76]. Thus, it is not surprising that dysfunction of miRNAs in malignancies is involved in cancer pathogenesis [77, 78], chemo/radiotherapy resistance [79, 80], tumor metastasis [81, 82], and other tumor-promoting mechanisms.

miRNAs are encoded throughout the human genome. Most miRNAs are found within intragenic regions as well as protein-coding or non-coding transcriptional units [83]. The production of miRNAs is a multistep process that is composed of three main events: cropping, export and dicing [84–87]. First, a miRNA gene is transcribed as primary capped and polyadenylated precursors of miRNA (pri-miRNA) by transcription factors and RNA polymerase II [88]. Then, these several kilobase-long transcripts are cleaved by the nuclear Drosha/DGCR8 heterodimer that releases a hairpin-structured pre-miRNA of 60–100 nucleotides [89]. Finally, Pre-miRNAs are cleaved into an 18–24 nucleotide mature miRNA duplex by the RNAseIII Dicer [90, 91]. One strand of the duplex (the guide strand of the miRNA) is then incorporated into the RNA-induced silencing complex (RISC) before annealing to its mRNA targets, whereas the other strand (the passenger strand) is degraded [92, 93].

As discussed previously, miRNA biogenesis is a complex multistep process, and each step of this process is tightly regulated. Mutation or aberrant expression of any component of the miRNA biogenesis machinery could contribute to a dysregulation in miRNA expression in tumors. The majority of miRNAs are transcribed by RNA polymerase II and III, and transcription factors significantly contribute to the activation of their expression by directly binding to the promoters of miRNAs. Recent evidence indicates that p63-mediated activation of miRNA transcription is an important event in the regulation of miRNA levels [94, 95].

### Transcriptional regulation of miRNAs by p63

Dysregulation of miRNA expression is common in human cancers. However, the mechanisms underlying miRNA dysregulation are not clear. As described above, transcriptional regulation is one of the most important steps in the synthesis of miRNAs. Transcriptional activation and repression of specific microRNAs by ΔNp63α was shown to attenuate the expression of several proteins involved in cell death and survival and to regulate tumor cell resistance to cisplatin [96]. In human bladder cancer cells, stable knockdown of ΔNp63α decreased binding of RNA Pol II to the miR-205 “host” gene (miR-205HG) promoter and reduced the expression of the primary and mature forms of miR-205 [97]. Finally, ΔNp63α decreased the levels of miR-205. Members of the miR-200 family are known regulators of cancer stem cells and epithelial-mesenchymal transitions. Knouf EC et al. found that p63 modulates the promoter activity of miRNAs of the miR-200 family by directly associating with the miR-200b/a/429 promoter, and p63 binding sites were significantly overrepresented among miRNA genes that were overexpressed in ovarian carcinoma [98]. In addition, p63 also proved to be an important regulator of apoptosis, proliferation, invasion, and metastasis in cancer cells by directly regulating the transcription of the miR-34 family [99], miR-138 and miR-181 [100].

Another important transcriptional program that has been identified involves post-transcriptional control of miRNAs by p63. The cropping step regulated by the Drosha/DGCR8 heterodimer is an important event in regulation of miRNA levels. There has been no direct study to prove that p63 can directly regulate the expression of Drosha. However, it has been reported that PY domain-containing proteins interact with WW domain-containing proteins. Interestingly, p63 contains a PY domain [101], while DGCR8 contains a WW domain [86], supporting the possibility of an interaction between p63 and DGCR8. Additionally, a study by Chakravarti D et al. showed that ΔNp63 is a transcriptional activator of DGCR8 [94]. Accumulating evidence has indicated that p63 can influence the ability of DGCR8 to process pre-miRNAs by regulating its expression [102]. As previously discussed, pre-miRNAs are cleaved in the cytoplasm near the terminal loop, a process that requires the RNAseIII Dicer. Several mechanisms can regulate Dicer expression, including the transcription factor p63. There are several TAp63 binding sites that have been identified within the Dicer promoter. Therefore, TAp63 can drive the expression of Dicer to influence the maturation of miRNAs (miR-31, miR-203, miR-130b, and miR-206) [103]. Huang Y et al's study suggests a synergistic effect of the Dicer-dependent miRNA (miR-630 and miR-885–3p) maturation after p-ΔNp63α-dependent miRNA transcriptional activation [104]. Interestingly, Boominathan L performed a study that investigated the relationship between p63 and the miRNA processing complex, and discovered that p63 was an important part of this complex during the maturation of miRNAs [105]. Furthermore, Boominathan L analyzed the promoter sequence of Drosha, DGCR8, Dicer and TARBP2 for p63 response elements (REs) and found several p63 and p53 REs in the promoter of a number of miRNA-processing components. He proposed a number of tumor suppressor pathways to illustrate p63 could function as both positive and negative regulators of miRNA through direct or indirect mechanisms [41]. Although the proposed pathways were not confirmed or validated by experimental studies, they provide a systemic overview of the interaction of the p53 family with miRNAs. Moreover, these two articles provide a new direction and methods for studying the interaction between p53 family and miRNAs, especially in the prediction of p53-REs and miRNA targets.

**Figure 2:**
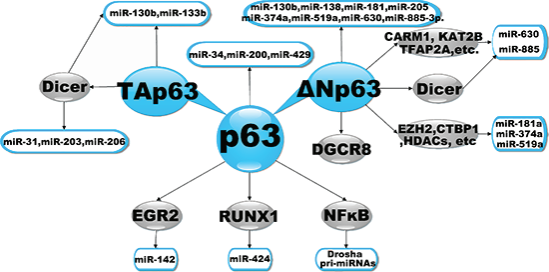
Transcriptional Regulation of miRNAs by p63 The diagram illustrates a simple schematic to highlight points of transcriptional regulation of miRNAs by p63. It illustrates that the p63 could modulate the expression of miRNAs by directly or alternative indirectly mechanisms.

Furthermore, p63 can control the expression of miRNAs by regulating the expression of miRNA transcription factors, as well as several key regulatory factors that allow for the correct maturation of primary miRNA. As an example of the former, the gene of the transcription factor early growth response 2 (EGR2) contains a p63 RE and is therefore a direct target of p63 [106]. Interestingly, it has recently been shown that EGR2 can bind to the pre-miR-142–3p promoter to regulate its expression [107]. Thus, it is plausible that p63 could regulate the expression of miR-142–3p through EGR2. Runt-related transcription factor 1 (RUNX1) has been identified as a direct target of p63 transcriptional regulation [108], and this transcription factor can regulate miR-424 expression [109]. Altogether, these findings suggest that p63 can promote the expression of miR-142–3p by regulating the transcription of RUNX1. Recently, the Drosha gene promoter has been shown to be a target of the NFκB transcription factor [110]. Another study showed that TAp63 can regulate NF-κB transcription and protein stability [111]. These data suggest that p63, by regulating the transcription of NF-κB, can promote the association between Drosha and pri-miRNAs. Moreover, it is reported that p-ΔNp63a is necessary to induce gene promoters for microRNAs (630 and 885–3p), together with certain transcriptional coactivators (e.g., CARM1, KAT2B, TFAP2A). Additionally, p-ΔNp63a, together with transcriptional corepressors (e.g., EZH2, CTBP1, HDACs), is needed to repress promoters for microRNAs (181a-5p, 374a-5p and 519a-3p) in SCC cells exposed to cisplatin [96]. These data suggest that p63, by regulating the transcription of miRNAs, plays important roles in various cancers (Figure 2). The miRNAs demonstrated to be regulated by p63 are summarized in Table 1.

**Table 1:** The miRNAs demonstrated to be transcriptional regulated by p63

miRNA Name	References
let7–5p	[128]
Let7i	[136]
miR-130b	[26, 100]
miR-133b	[131]
miR-138	[100]
miR-155	[63]
miR-181	[96, 100, 104, 130]
miR-185–5p	[128]
miR-193a-5p	[138]
miR-200	[98]
miR-205	[27, 97]
miR-297	[96]
miR-34	[99]
miR-374a	[104, 130]
miR-429	[98]
miR-485–5p	[96]
miR-519a	[104, 130]
miR-630	[104, 130]
miR-885–3p	[104, 130]
miR-92b-3p	[96]

### Regulation of p63 by miRNAs

As discussed above, p63 has been defined as a regulator of miRNAs. Conversely, p63 expression can also be controlled by specific miRNAs in certain tissues. Recent studies identified p63 as one of the conserved targets of miR-203 [112–114], and regulation of the expression of ΔNp63 is an important molecular step for miR-203-induced suppression of proliferation and cell-cycle arrest [115–117]. Lena AM et al. confirmed these findings and noted that miR-203 can regulate ΔNp63 levels in head and neck squamous cell carcinoma [118] and significantly suppress the proliferation and migration of these cancer cells [119–120]. In addition, a study by Manni I et al. demonstrated that p63 is a direct target of miR-92, and concluded that negative regulation of ΔNp63 expression is one of the molecular mechanisms through which miR-92 inhibits cell proliferation [123].

Additionally, Papagiannakopoulos T et al. showed for the first time that miR-21 targets the tumor-suppressive protein TAp63, and confirmed that miR-21 is necessary for suppression of apoptosis in glioblastoma cells [121]. Wang T et al. found that TAp63 was a target of miR-21 in HaCaT cells, and this miRNA could regulate the response of epithelial cells to TGF-β with a potential impact on tumorigenesis [122]. Furthermore, researchers demonstrated that both miR-21 and miR-30b/c can target the 3′UTR of TAp63 mRNA and that TAp63 proteins mediate some of the effects of miR-21 and miR-30 on tumor necrosis factor-related apoptosis-inducing ligand (TRAIL) resistance in primary human glioblastoma cells and lung cancer cells [124]. Endogenous miR-302 was also proven to reduce p63 protein and mRNA levels through two target sites within the p63 3′UTR in testicular cancer cells, indicating that miR-302 may counteract apoptosis [125]. The miRNAs that have been demonstrated to regulate p63 are listed in Table 2.

**Table 2:** The miRNAs demonstrated to regulate p63

miRNA name	References
miR-130b	[100]
miR-181a	[130]
miR-196a2*	[137]
miR-203	[112–120]
miR-21	[121, 122]
miR-30b/c	[124]
miR-302	[125]
miR-374a	[130]
miR-519a	[130]
miR-630	[130]
miR-885–3p	[130]
miR-92	[123]

**Figure 3:**
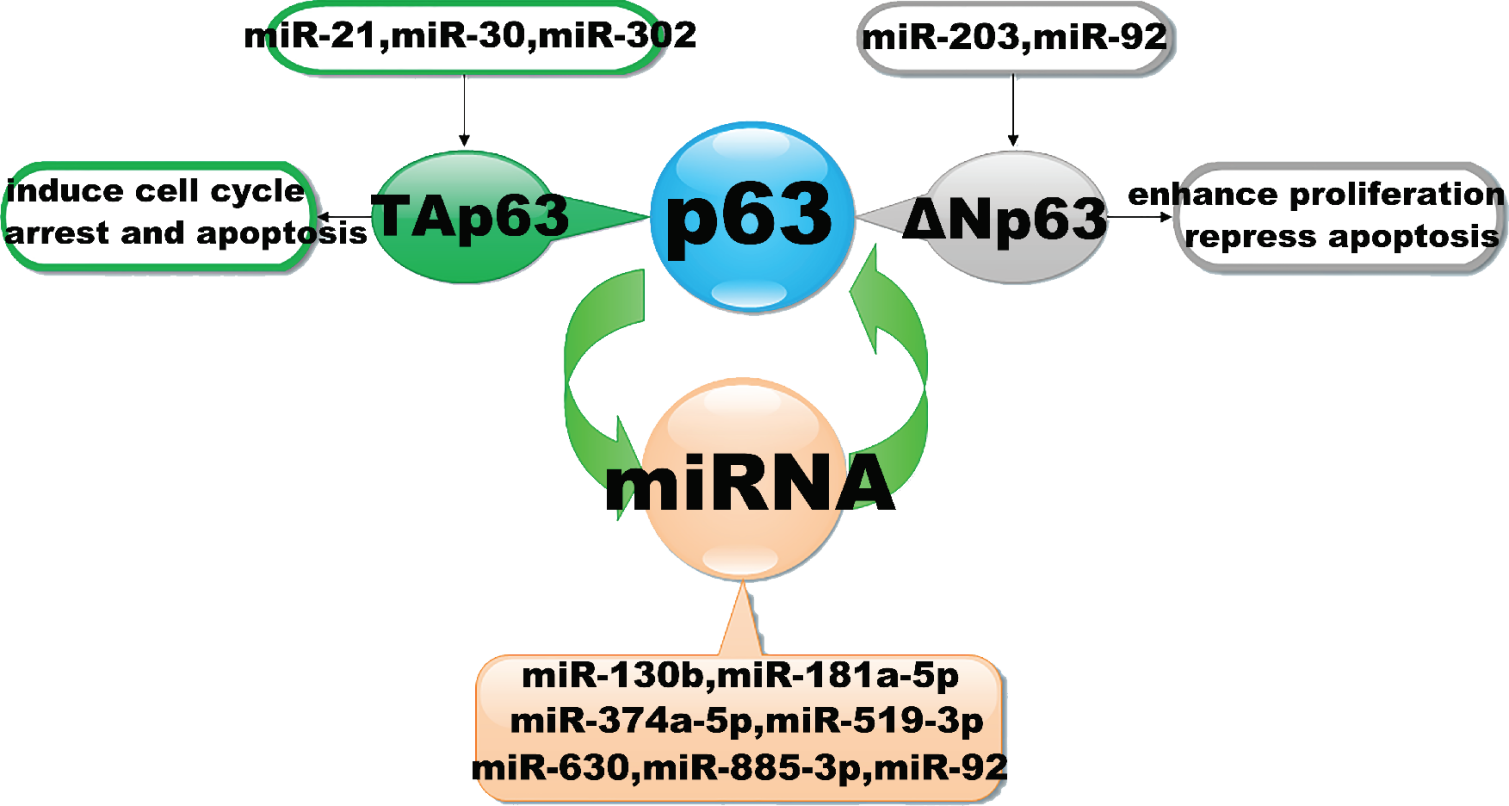
Interaction of miRNAs with the p63 signalling pathway The diagram schematic represented the regulatory loops that exist between p63 and miRNAs. It illustrates that miRNAs could regulate the expression by targeting to p63. Conversely, miRNAs expression also could be inhibited by p63.

Given these findings, p63 isoforms regulated by miRNAs can be divided into two classes: ΔNp63-isoforms, which enhance cell proliferation and repress apoptosis and are repressed by miR-92 and miR-203, and Tap63-isoforms, which induce cell cycle arrest and apoptosis and are repressed by miR-21, miR-30 and miR-302 (Figure 3).

As shown by the above examples, an emerging consensus is that the transcription factor p63 is particularly prominent within regulatory circuits controlling the expression and function of miRNAs. It has been reported that ΔNp63 can inhibit miR-130b expression by binding directly to p63-Res located in close proximity to the genomic locus of miR-130b. Conversely, ΔNp63 expression can also be inhibited by miR-130b [100]. Thus, it is plausible that p63 and miR-130b are able to regulate one another to form a p63/miR-130b autoregulatory feedback loop [100]. In addition, p63 and miR-92 have been shown to form a p63/miRNA autoregulatory feedback loop [96, 123]. Phosphorylation is a prevalent mechanism by which the activity of transcription factors is regulated rapidly in response to changes in the cellular environment, and it provides dynamic and precise tuning of their transactivation potential. Accumulated evidence suggests that the phosphorylated-ΔNp63α (p-ΔNp63α) transcription factor is indispensable for activation and inhibition of the expression of specific genes [126, 127]. As the expression of miRNAs is consistently maintained by RNA polymerase II and III transcriptional machinery, the regulatory role of p-ΔNp63α in the miRNA maturation process is highly evident [128, 129]. Indeed, Huang Y et al. demonstrated that p-ΔNp63α can transcriptionally regulate microRNA gene promoters, while total ΔNp63α levels and p-ΔNp63α levels are maintained by miRNAs [130]. P-ΔNp63α/microRNA feedback regulation plays a key role in squamous carcinoma cells exposed to cisplatin, thereby revealing a regulatory feedback loop between p63 and miRNAs (Figure 3) [96]. These results prompted us to analyze miRNA binding to the 3′UTR of p63 via multiple components, including miRtarBase, Targetscan,microRNA.ORG, DIANA-MICROT, RNA22-HSA and miRDB. Table 3 refers to miRNAs that are predicted to target p63 mRNA by at least two different algorithms. Because p63 can bind to p53 consensus sequences [99], we screened a region of ~2000 bp upstream of the miRNAs listed in Table 3 and searched transcriptional start sites for p53/p63 Res using the JASPAR (profile score threshold 80%) and TFSEARCH datasets. Remarkably, bioinformatic analyses revealed many p53- and p63-Res in the promoters of miRNAs, indicating that they could be direct transcriptional targets of p63 (Table 4). In support of this data, we screened a region of ~2000 bp upstream of the miR-133b transcriptional start site for p53RE. We showed that Tap63 could directly transcriptionally regulate miR-133b [131]. Collectively, these findings suggest a feed-forward loop, where p63 expression suppresses miRNAs, as well as a negative feedback loop, where p63 expression regulates via its own 3′UTR and miRNAs. Taken together, the molecular interrelationship between p63 and specific miRNAs is a key functional network in tumorigenesis. Although we are only beginning to uncover their complexity, such circuits will occupy a particularly important role within the relationship between p63 and miRNAs.

**Table 3:** Identified miRNAs targeting the 3ʹUTR of p63 mRNA by web algorithms

miRNA	Sequence	algorithms
hsa-miR-1297	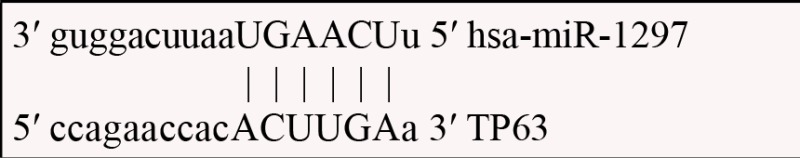	microRNA.ORG Targetscan
hsa-miR-130a	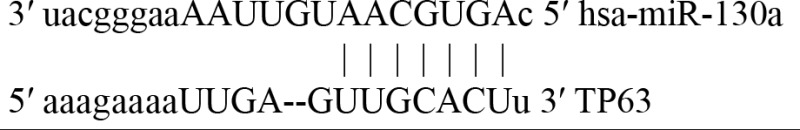	microRNA.ORG Targetscan DIANA-MICROT
hsa-miR-130b	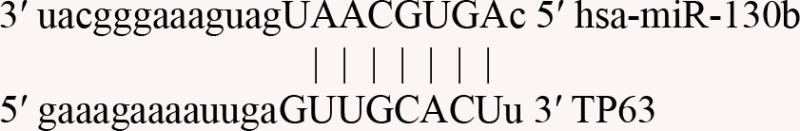	microRNA.ORG Targetscan DIANA-MICROT
hsa-miR-133b	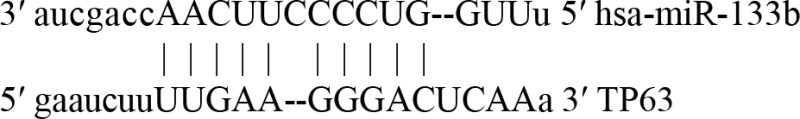	microRNA.ORG RNA22-HSA
hsa-miR-136	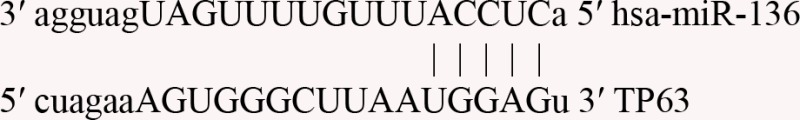	microRNA.ORG miRDB
hsa-miR-140	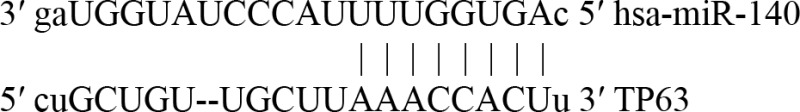	microRNA.ORG DIANA-MICROT
hsa-miR-149	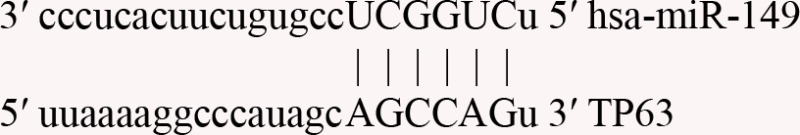	microRNA.ORG DIANA-MICROT
hsa-miR-181a	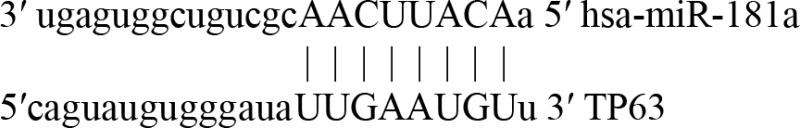	microRNA.ORG miRDB
hsa-miR-181b	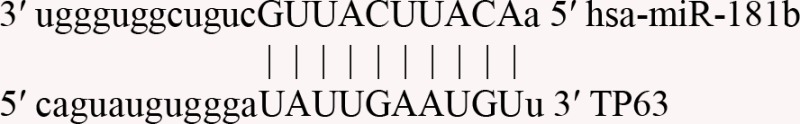	microRNA.ORG miRDB
hsa-miR-181c	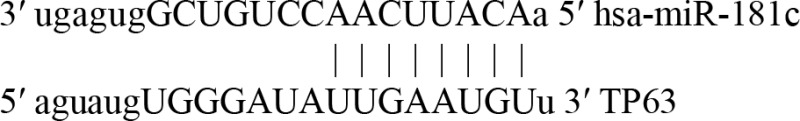	microRNA.ORG miRDB
hsa-miR-181d	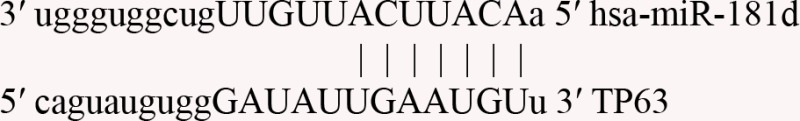	microRNA.ORG miRDB
hsa-miR-186	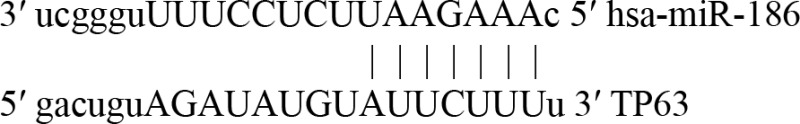	microRNA.ORG miRDB
hsa-miR-203	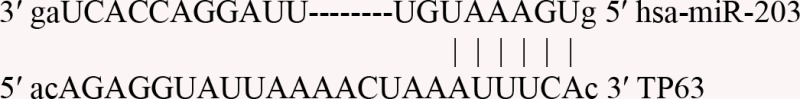	microRNA.ORG miRtarBase
hsa-miR-204	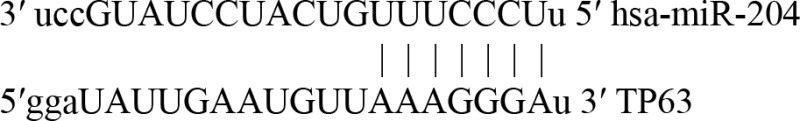	microRNA.ORG miRDB
hsa-miR-21	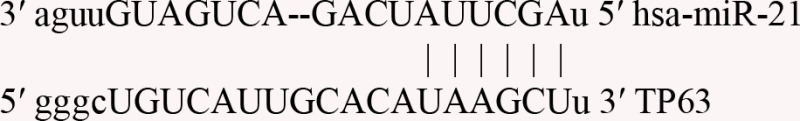	microRNA.ORG miRtarBase
hsa-miR-211	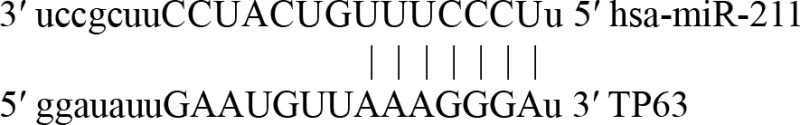	microRNA.ORG miRDB
hsa-miR-221	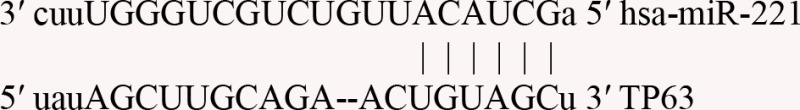	microRNA.ORG DIANA-MICROT
hsa-miR-223	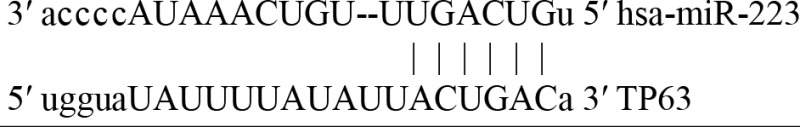	microRNA.ORG miRDB
hsa-miR-224	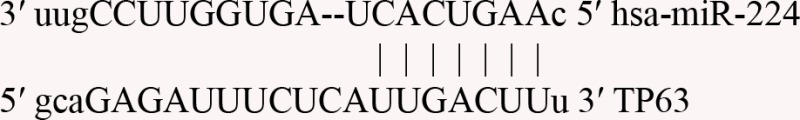	microRNA.ORG DIANA-MICROT
hsa-miR-23a	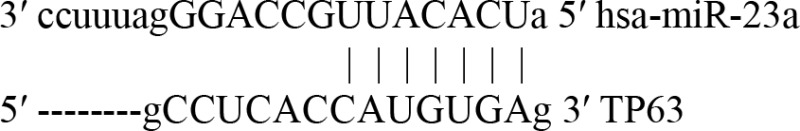	microRNA.ORG miRDB
hsa-miR-23b	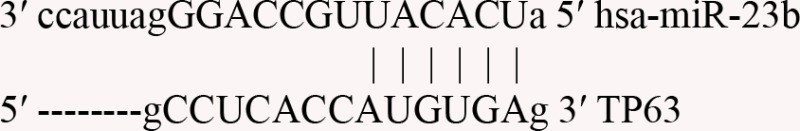	microRNA.ORG miRDB
hsa-miR-26a	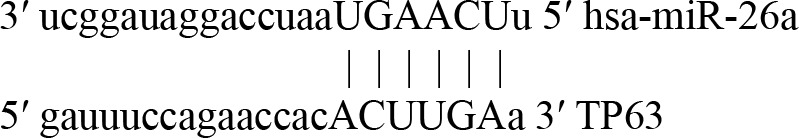	microRNA.ORG Targetscan
hsa-miR-26b	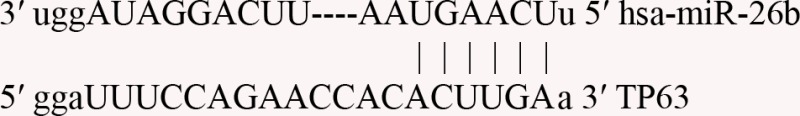	microRNA.ORG Targetscan
hsa-miR-30a	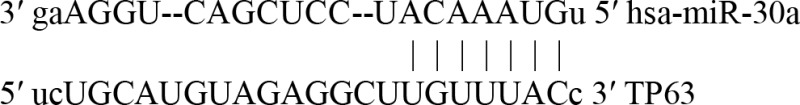	microRNA.ORG miRDB
hsa-miR-30b	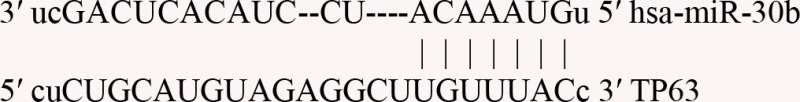	microRNA.ORG miRDB
hsa-miR-30c	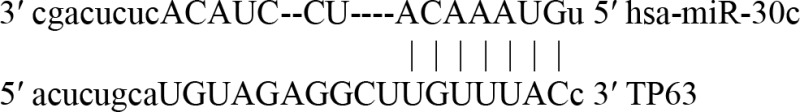	microRNA.ORG miRDB
hsa-miR-30d	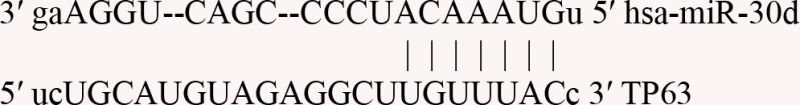	microRNA.ORG miRDB
hsa-miR-30e	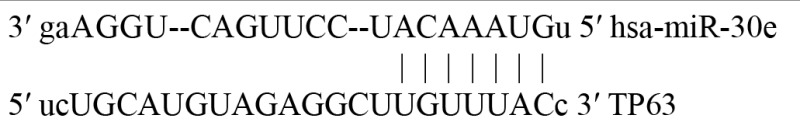	microRNA.ORG miRDB
hsa-miR-301a	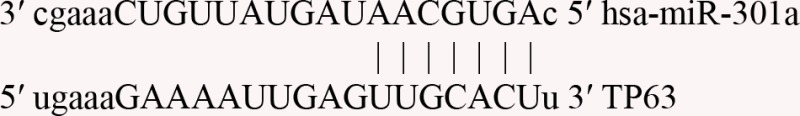	microRNA.ORG Targetscan
hsa-miR-301b	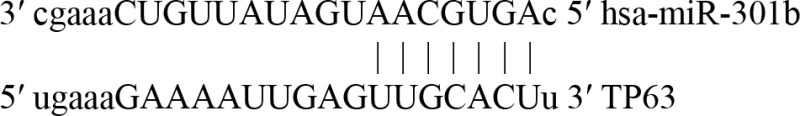	microRNA.ORG Targetscan
hsa-miR-302a	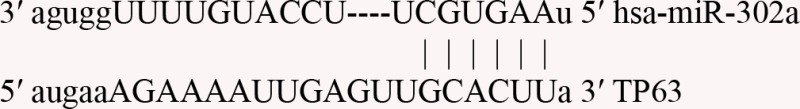	microRNA.ORG miRtarBase DIANA-MICROT
hsa-miR-302b	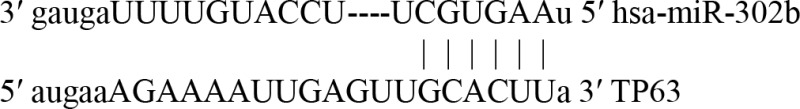	microRNA.ORG miRtarBase DIANA-MICROT
hsa-miR-302c	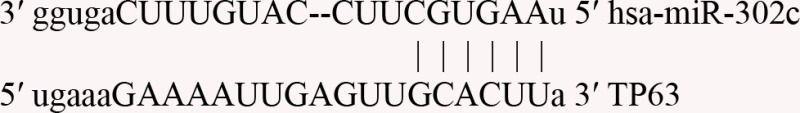	microRNA.ORG miRtarBase DIANA-MICROT
hsa-miR-302d	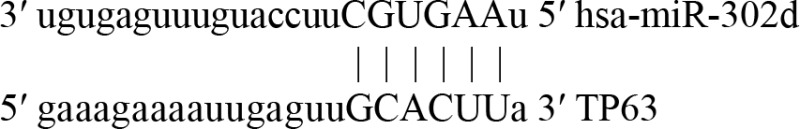	microRNA.ORG miRtarBase DIANA-MICROT
hsa-miR-302e	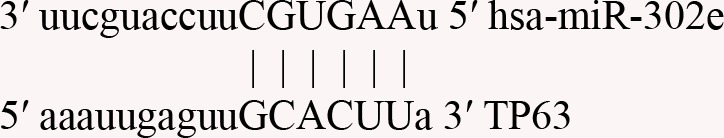	microRNA.ORG miRtarBase DIANA-MICROT
hsa-miR-3163	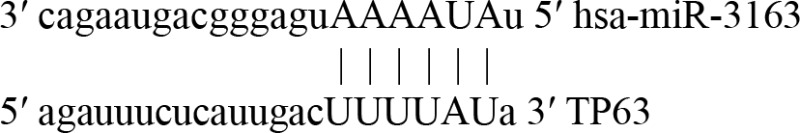	miRDB DIANA-MICROT
hsa-miR-32	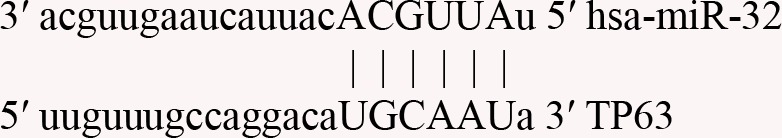	microRNA.ORG DIANA-MICROT
hsa-miR-340	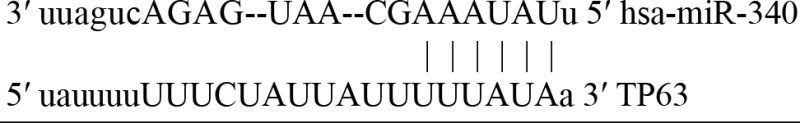	microRNA.ORG DIANA-MICROT
hsa-miR-3666	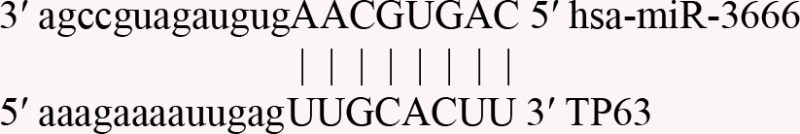	Targetscan DIANA-MICROT
hsa-miR-371	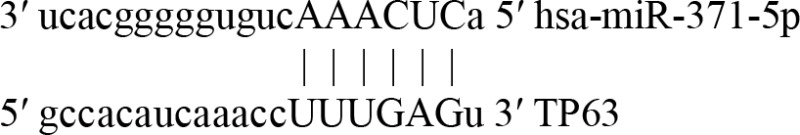	microRNA.ORG DIANA-MICROT
hsa-miR-374a	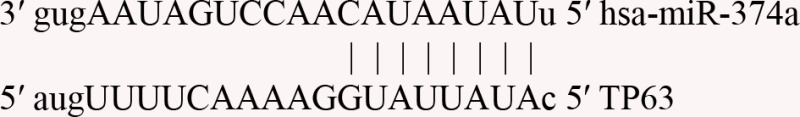	microRNA.ORG DIANA-MICROT
hsa-miR-374b	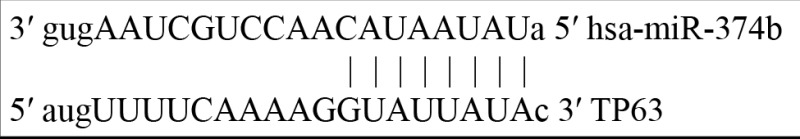	microRNA.ORG DIANA-MICROT
hsa-miR-377	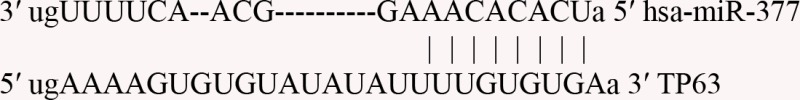	microRNA.ORG DIANA-MICROT
hsa-miR-454	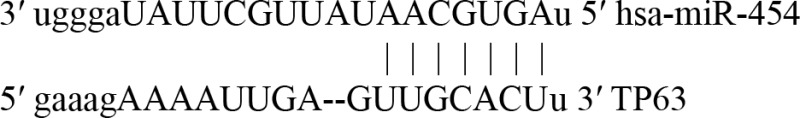	microRNA.ORG Targetscan
hsa-miR-495	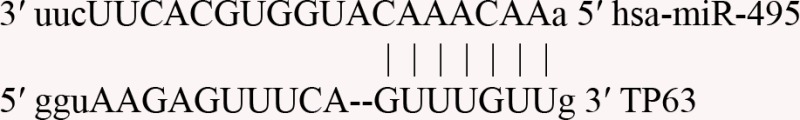	microRNA.ORG DIANA-MICROT
hsa-mir-519a-3p	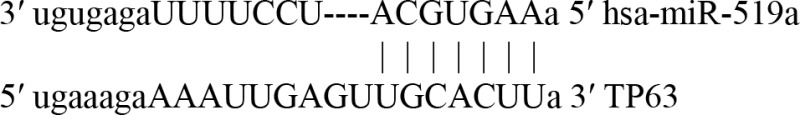	microRNA.ORG Targetscan miRDB
hsa-mir-519b-3p	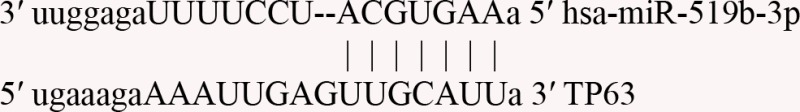	microRNA.ORG Targetscan miRDB
hsa-mir-519c-3p	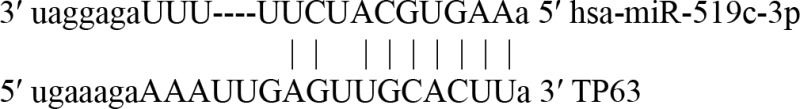	microRNA.ORG Targetscan miRDB
hsa-mir-519e	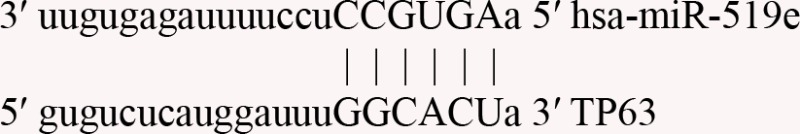	microRNA.ORG miRDB
hsa-miR-520a	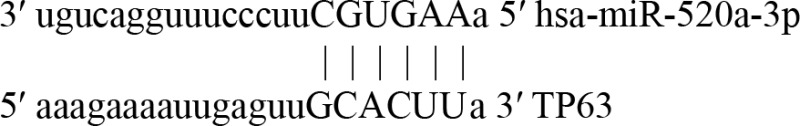	microRNA.ORG miRDB
hsa-miR-520b	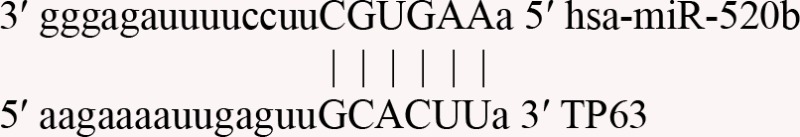	microRNA.ORG miRDB
hsa-miR-520c	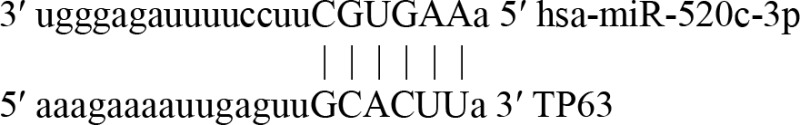	microRNA.ORG miRDB
hsa-miR-520d	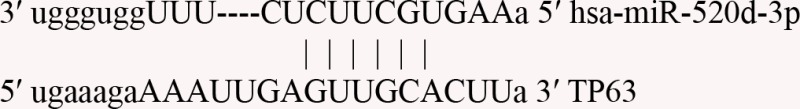	microRNA.ORG miRDB
hsa-miR-520e	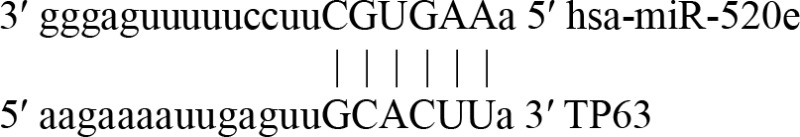	microRNA.ORG miRDB
hsa-miR-524	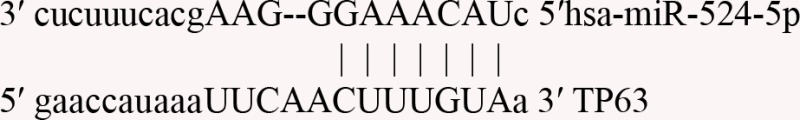	miRDB DIANA-MICROT
hsa-miR-539	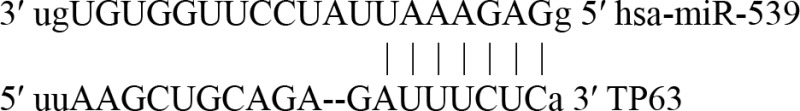	microRNA.ORG DIANA-MICROT
hsa-miR-543	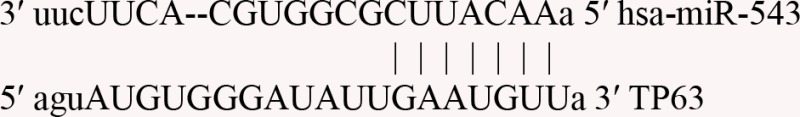	microRNA.ORG miRDB
hsa-miR-590-3p	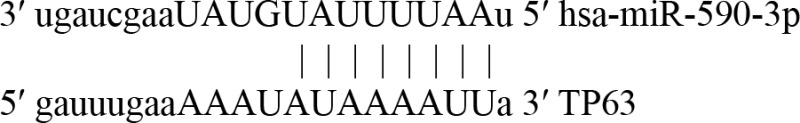	microRNA.ORG miRDB
hsa-miR-630	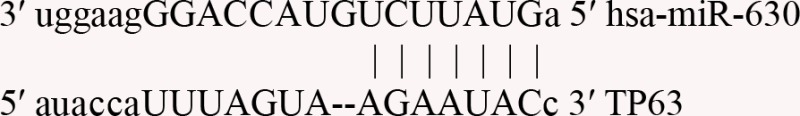	RNA22-HSA
hsa-miR-875	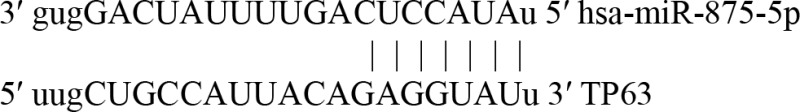	microRNA.ORG miRDB
hsa-miR-885-3p	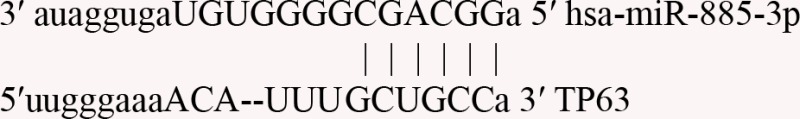	RNA22-HSA microRNA.ORG
hsa-miR-92a	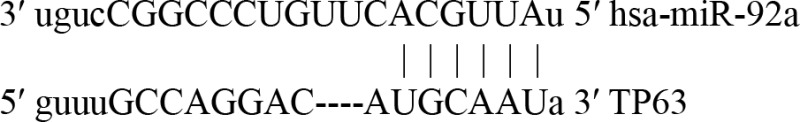	microRNA.ORG miRtarBase DIANA-MICROT
hsa-miR-92b	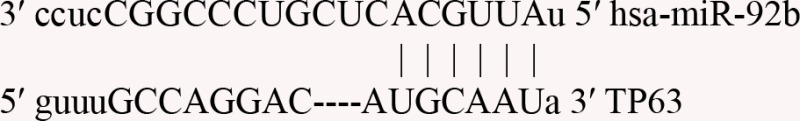	microRNA.ORG miRtarBase DIANA-MICROT

**Table 4:** The promoters of miRNAs contain p53/p63-REs

miRNAName	TFSEARCH p53/p63-REs	JASPAR datasetsp53/p63-REs	predicted site sequence
	Scores	Scores	
hsa-miR-1297	67.1	10.384	ATTCATGTGCTCACCAGCAT
hsa-miR-130a	62.2	10.215	GGTCCTGTCCTCCCATGCCA
		8.413	GGGGAGGCACTGGCAGGCCT
		6.776	GGCCCCGCCCCAGCCAGCCT
hsa-miR-130b	None	None	None
hsa-miR-133b	63.8	7.731	CCCCCTGCTCTGGCTGGTCA
hsa-miR-136	60.9	7.980	AGCCTGGCTCTTTCTTGCAT
		6.143	ATCCTGGCACCCACAGGTTC
hsa-miR-140	62.9	7.719	ATGCCTGTTCATACAGACAG
		6.521	CCTCCCGCCCCTGCCTGCTG
		6.205	TGGTAGGTTACGTCATGCTG
hsa-miR-149	70	7.225	TGAGATGCCGTGGCCGGTCC
hsa-miR-181a	60.9	11.661	TTACATGTGCCACCCTGCCT
		11.511	ATGTGTGCTCAAACTTGCTT
hsa-miR-181b	60.9	8.519	AAACAGGACGTAGCAAGTAA
		6.827	TTACTAGTGCCCACATATCC
		6.616	TCTAATGTACCTACATGTCT
		6.382	TTACAGGTACTAATATGCAA
hsa-miR-181c	67.8	9.772	AGGCCAGCACTCCCCTGCAC
hsa-miR-181d	67.8	9.772	AGGCCAGCACTCCCCTGCAC
		6.779	AATCCAGCCTGGGCACGTCC
hsa-miR-186	None	None	None
hsa-miR-203	63.8	10.527	GCGCTGGTCCTCACCTGTTC
		6.155	GGGTGTGTCCAGCCCAGCCC
hsa-miR-204	60.9	None	None
hsa-miR-21	60.6	None	None
hsa-miR-211	60.9	None	None
hsa-miR-221	61.6	8.541	GTACCAGTGCACTCCAGCCT
hsa-miR-223	None	None	None
hsa-miR-224	61.6	7.065	GCCCCTGCCCATCCAAGCTC
hsa-miR-23a	70.7	10.796	AGCCATGATCACACCAGCCT
hsa-miR-23b	61.2	7.873	ATTTTTGCCCAGGCAGGCAA
hsa-miR-26a	None	6.504	AGGCATGCTTCATCATCCTC
hsa-miR-26b	61.8	8.511	ACACCTGGGCACACATGCAG
		6.475	ATGCAGGATTCTGCAGGCCA
		6.258	CGGCAAGATCCTCCTGGCTC
hsa-miR-30a	66.8	8.706	AAAAATGTACAGACATGGTT
		7.945	TCTCATGGCCCAGCATGACT
hsa-miR-30b	61.2	8.073	ATTCAAGTAGATCCCTGCCA
		8.032	GAGCATACAGACACTTGCCA
		7.230	AGGCAAGAGCATACAGACAC
hsa-miR-30c	60.3	8.774	ATGCAAGTGCAAAAATGTAT
hsa-miR-30d	None	None	None
hsa-miR-30e	69.7	None	None
hsa-miR-301a	64.2	None	None
hsa-miR-301b	70.7	None	None
hsa-miR-302a	62.5	10.060	AAGCAAGTACATCCACGTTT
		9.027	AAATAAGCCCATTCCAGCCT
		8.869	CAGCAAGTGCCTCCATGTTA
		7.824	TGTCATGTCACAGCAAGTGC
hsa-miR-302b	None	10.060	AAGCAAGTACATCCACGTTT
		9.027	AAATAAGCCCATTCCAGCCT
		8.869	CAGCAAGTGCCTCCATGTTA
		8.339	CAGCAGGTACCCCCATGTTA
		7.824	TGTCATGTCACAGCAAGTGC
hsa-miR-302c	62.5	10.060	AAGCAAGTACATCCACGTTT
		9.027	AAATAAGCCCATTCCAGCCT
		8.869	CAGCAAGTGCCTCCATGTTA
		8.339	CAGCAGGTACCCCCATGTTA
		7.824	TGTCATGTCACAGCAAGTGC
hsa-miR-302d	None	None	None
hsa-miR-302e	None	None	None
hsa-miR-3163	None	None	None
hsa-miR-32	None	None	None
hsa-miR-340	64.8	8.837	CTGCCGGTGGCAACATGTAG
		7.456	AGACAGGTCCAGGCTTCAAC
		7.255	AGGCATGGTGGCACATGTCT
		7.034	CACCAAGTAGGAACATGTAA
		6.992	TCTTATGTCCAGACTTGAGT
hsa-miR-3666	None	None	None
hsa-miR-371	60.9	None	None
hsa-miR-374a	62.2	12.317	AAACATGTCTTAGCTGGCTT
hsa-miR-374b	60.3	6.258	AGGCATGTGCCACCACACCT
hsa-miR-377	68.4	10.889	GTCCATGACCAACCATGTTC
		6.431	GGTCGTGCACCTGCAGGCGT
hsa-miR-454	None	8.404	TGGCCAGTACTGGCTTATTA
		7.502	AGGCGTGCACCACCATACCC
hsa-miR-495	66.1	8.931	AGGCAAGGAGATGCTTGCTG
		7.293	TTACTTGTTTAAGCCAGTTG
hsa-miR-519a-3p	None	None	None
hsa-miR-519b-3p	None	None	None
hsa-miR-519c-3p	None	None	None
hsa-miR-519e	None	None	None
hsa-miR-520a	None	None	None
hsa-miR-520b	66.4	15.207	AGGCATGTGCTACCATGCCC
		7.330	AGGCATGCACCACCACACCT
		7.330	AGGCATGCACCACCACACCT
		6.271	TTTTTTGTACAGACATGGTT
hsa-miR-520c	60.4	11.688	TCTCAGGTTCAAGCAAGTCT
hsa-miR-520d	None	None	None
hsa-miR-520e	61.6	8.184	AGGCGTGAGCTACCCTGCCC
		7.625	GCTCAAGCATCCTCCTGCCT
		7.028	TGGTGTGCAGTGTCATGTTC
hsa-miR-524	66.4	11.360	AGGCACGCACCGCCATGCCG
		8.473	GCGCAGGTGCACTCCAGCCT
		6.384	TTACAAGCACCCACCAACAC
hsa-miR-539	64.2	11.098	GAACGAGTTAAGACTTGTAC
		7.204	CACCACGCACACACAGGCAT
		6.519	GGGCAAGGGCTGGCATGGAG
hsa-miR-543	62.5	14.784	ACGCCTGCCCTGTCCTGCAC
hsa-miR-590	64.2	9.773	CAGGGTGCCCAGGCATGCAA
hsa-miR-630	None	None	None
hsa-miR-875	None	7.726	CAGCCAGCCCCCTCATATCC
hsa-miR-885-3p	None	None	None
hsa-miR-92a	70.7	12.995	GCACTTGTCCCGGCCTGTTG
		8.804	ACTCCAGCTTCGGCCTGTCG
		7.105	GAGCTTATTTAGACATGTAT
		6.601	TGACAAGTTCATTCTTCTCT
		6.189	ATGCAATTCCTTACCTGTAA
hsa-miR-92b	60.6	7.510	GTTCAAGACCAGCCTGGCCA

### Conclusion and future perspective

It is well established that p63 plays an important role in the development and progression of cancer. Accordingly, p63 functions within a wide biological spectrum, stretching from epidermal mesenchymal transition [132] to senescence, cell death and cell cycle arrest [133]. These states are all determinants in cancer progression, and thus p63 affects the chemosensitivity of tumors [134]. In addition, p63 is deregulated in tumor tissues, and there has been much debate as to whether p63 behaves as a tumor suppressor gene or an oncogene, as current studies support both sides. The P63 gene encodes two major protein isoforms, TAp63 and ΔNp63, which have opposing regulatory functions on downstream target genes [135, 136]. Previous studies have shown that p63, by regulating the transcription of miRNAs, contributes to multiple mechanisms implicated in the development and progression of cancer. The diverse actions of these miRNAs affected by TAp63 isoforms and ΔNp63 isoforms further complicate the tumor cell response. The former generally act as pro-apoptotic and tumor suppressive agents, while the latter function more as an anti-apoptotic and oncogenic factors.

miRNAs are small, non-coding RNAs that typically inhibit the translation and stability of mRNAs. Over 60% of human protein-coding genes are predicted to be controlled by miRNAs. It is clear that miRNAs provide a complicated regulation of the signaling networks involved in cellular processes such as differentiation, stress response, cell cycle regulation, apoptosis, and migration. Thus, miRNA dysregulation has been shown to play an essential role in the development and progression of cancer. Furthermore, miRNAs are able to target the p63 gene, a key regulator of the surveillance network, potentially providing a new means of perturbing p63 signaling networks in cancer. The components of the miRNA network consist of hundreds of protein-coding genes, including those acting upstream to regulate miRNA expression, those functioning downstream to mediate miRNA effects, and those forming regulatory feedback loops.

Our review covers the p63/miRNA autoregulatory feedback loop. There are, in total, approximately 39 pairs of p63-miRNA feedback predicted. Four of these (ΔNp63/miR-130b, ΔNp63/miR-92, ΔNp63/miR-181a-5p and ΔNp63/miR-374a-5p) have been validated [96, 100, 123, 130]. A study by Huang et al. revealed and validated three additional p63-miRNA feedback loops (ΔNp63/miR-519a-3p, ΔNp63/miR-630 and ΔNp63/miR-885–3p). Interestingly, we were unable to find the p53/p63-Res showing that 63 binds to the promoter of these three miRNAs (miR-519a-3p, miR-630 and miR-885–3p) in the JASPAR (profile score threshold 80%) datasets. Although very little is currently known about the p63/miRNA autoregulatory feedback loop, it is likely that it plays an important role in tumorigenesis. Moreover, the expression of p63 and miRNAs, as well as the isoforms of p63 that are predominately present, will vary for individual tumors, and it remains to be seen how dependent these relationships are on cancer progress. It has become increasingly clear that p63 and miRNAs are linked in a novel autoregulatory feedback loop that controls tumorigenesis.

It is now apparent that many tumors are dependent upon a dysregulation of p63 and miRNAs, providing a rationale for therapeutic intervention of p63 and miRNAs. Therefore, the p63/miRNA network represents a novel approach for treatment and is likely to lead to new therapeutic anticancer strategies.
